# Average duration of prior treatment lines predicts clinical benefit to eribulin chemotherapy in patients with metastatic breast cancer

**DOI:** 10.1007/s10549-021-06438-7

**Published:** 2021-11-29

**Authors:** Faye Coe, Vivek Misra, Yamini McCabe, Helen Adderley, Laura Woodhouse, Zaheen Ayub, Xin Wang, Sacha Howell, Maria Ekholm

**Affiliations:** 1 Department of Pharmacy, The Christie NHS Foundation Trust, Manchester, UK; 2 Department of Clinical Oncology, The Christie NHS Foundation Trust, Manchester, UK; 3 Department of Medical Oncology, The Christie NHS Foundation Trust, Manchester, UK; 4 Department of Analytics and Statistics, Digital Services, The Christie NHS Foundation Trust, Manchester, UK; 5 Department of Oncology, Ryhov Hospital, Jönköping, Sweden; 6 Institute of Biomedicine, Department of Laboratory Medicine, Sahlgrenska Center for Cancer Research, Sahlgrenska Academy at University of Gothenburg, Gothenburg, Sweden

**Keywords:** Metastatic breast cancer, Eribulin, Real world, Subtypes, Chemotherapy, Palliative

## Abstract

**Purpose:**

The aim of this study was to identify factors associated with progression-free survival (PFS) and overall survival (OS) in patients with metastatic breast cancer (MBC) treated with eribulin in a real-world setting, to improve information provision in those considering treatment.

**Methods:**

Patients treated with eribulin for MBC at The Christie NHS Foundation Trust, Manchester, UK, between August 2011 and December 2018 were included (*n* = 439). Data were collected by retrospective review of medical records and electronic prescribing systems. Factors such as biological subtype, distant recurrence-free interval, previous lines of chemotherapy and the ‘average duration of previous treatment lines’ (ADPT) (calculated as: (date of initiation of eribulin–date of MBC) / the number of previous treatment lines in the metastatic setting) were evaluated for prognostic impact using Cox proportional hazards regression.

**Results:**

In the full cohort, the median PFS and OS were 4.1 months (95% CI 3.7–4.4) and 8.6 months (95% CI 7.4–9.8), respectively. Outcomes were significantly inferior for those with triple-negative breast cancer (TNBC) (*n* = 92); PFS_TNBC_: 2.4 months (95% CI 2.1–3.0), *p* =  < 0.001 and OS_TNBC_: 5.4 months (95% CI 4.6–6.6), *p* =  < 0.001. ADPT was the only factor other than subtype significantly associated with PFS and OS. Longer ADPT was also significantly associated with PFS and OS in those with TNBC. For example, women in the lowest ADPT tertile (< 5.0 months) achieved a median OS of only 4.3 months, whereas those in the upper ADPT tertile (> 8.7 months) had a median OS of 12.1 months (*p* = 0.004).

**Conclusion:**

Our results indicate that the ADPT lines is an important factor when predicting the outcome with eribulin chemotherapy in a palliative setting and that quantitative guidance on the likely PFS and OS with treatment can be provided using ADPT. Validation in additional cohorts is warranted.

## Introduction

Breast cancer is the most common cancer in women with approximately 1.7 million new cases per year worldwide [1]. The aim of systemic therapy for metastatic breast cancer (MBC) is to delay disease progression, improve overall survival (OS) and at the same time maintain or improve the quality of life by controlling cancer-related symptoms. Although clear guidelines exist for first-line treatment options, later treatment lines are less well evidenced, potentially less efficacious and therefore the side effect profile and patient wishes are highly important to take into consideration when choosing treatment [2].

Eribulin, a non-taxane microtubule dynamics inhibitor, was approved for the treatment of advanced breast cancer based on two phase III studies [3, 4]. In EMBRACE, heavily pretreated patients with MBC of all subtypes were randomised 2:1 to receive eribulin or treatment of physician’s choice (TPC) [3]. Progression-free survival (PFS) was 3.7 months in the eribulin arm and eribulin was shown to improve OS significantly compared to TPC (13.1 months vs. 10.6 months; Hazard Ratio (HR) = 0.81, *p* = 0.041) [3]. In Study 301, patients with MBC of all subtypes previously treated with anthracyclines and taxanes were assigned 1:1 to receive eribulin or capecitabine as 1st-, 2nd- or 3rd-line therapy. In this trial, PFS was 4.1 months and no significant difference in OS was seen between the 2 groups (15.9 months vs. 14.5 months, *p* = 0.056) [4]. However, a subgroup analysis of patients with human epidermal growth factor receptor 2 (HER2) negative MBC did show a longer OS in the eribulin arm (16.1 months vs. 13.5 months, *p* = 0.026) [5]. A pooled analysis of EMBRACE and Study 301 has also been published (*n* = 1864; eribulin *n* = 1062, TPC or capecitabine *n* = 802) with OS data favouring eribulin (15.2 months vs. 12.8 months, *p* = 0.003) and a similar positive effect seen across the different subtypes [6].

The primary aim of the current study was to identify factors associated with PFS and OS in patients with different subtypes of MBC treated with eribulin in a real-world setting to improve information provision to patients considering palliative treatment in the metastatic setting.

## Methods

### Patients

All patients treated with eribulin for MBC at The Christie NHS Foundation Trust (The Christie), Manchester, UK, between 1st August 2011 and 31st December 2018 were identified through the electronic patient data systems. Medical records and electronic prescribing systems were reviewed by four doctors (ME, YM, LW and HA) and two pharmacists (FC and ZA). Data on breast cancer history and previous treatments, patient and tumour characteristics, eribulin treatment, hospital admissions and outcome were collected using a predefined case report form. Last date for follow-up was 30th of March 2020. Detailed information on adverse events was not collected. The majority of patients were treated with the standard dose of eribulin, i.e. 1.23 mg/m^2^ on days 1 and 8 in a 21-day cycle; however, but those who recieved dose reductions from cycle 1 are included. Radiological assessment was performed as per standard of care, generally every 4 cycles. Patients were divided into the following subtypes based on the biological characteristics of the tumour; oestrogen receptor (ER) positive and/or progesterone receptor (PR) positive and HER2 negative (ER + /HER2-); HER2 positive, irrespective of ER/PR status (HER2 +); ER-/PR-/HER2- (TNBC). ER/PR positivity was defined as ≥ 1% positively stained nuclei or quick score (QS) ≥ 3 if no percentage had been recorded.

### Statistical analyses

Summary statistics were provided for patient and tumour characteristics. Median Tests were applied for continuous variables and Chi-squared Tests were applied for categorical variables to assess the differences between subtype groups for corresponding variables. PFS was defined as the time from start of eribulin treatment until progressive disease, primarily radiological but clinical for patients who clearly had progressive disease without undergoing a scan, or death from any cause. OS was defined as the time from start of treatment until death from any cause. Patients lost to follow-up were censored at the day of their last eribulin treatment for PFS and the day of their last follow-up for OS. Distant recurrence-free interval (DRFi) was defined as the date of the primary cancer until diagnosis of distant recurrence. The ‘average duration of previous treatment lines’ (ADPT) was calculated as: (date of initiation of eribulin–date of MBC) / the number of previous treatment lines in the metastatic setting, including endocrine therapy for patients with ER + disease. Treatments given < 1 month, irrespectively of stop cause, was disregarded when counting the number of previous treatment lines. The Kaplan–Meier method was used to estimate median survival and corresponding 95% confidence intervals (CIs). Univariable Cox proportional hazards (PH) regression was applied to assess the association between PFS and OS, respectively, and DRFi, number of previous chemotherapy lines for MBC, and ADPT categorised as tertiles for the whole group and subgroups separately and as continuous variables. Patients with de novo metastatic disease were treated as ‘no record’ in the calculation of DRFi and they were excluded when calculating the tertiles for DRFi. Patients who received eribulin as first line (because of recurrence shortly after having completed adjuvant therapy, including an anthracycline and a taxane) were treated as ‘no record’ in the calculation of ADPT and they were excluded from the calculation of the tertiles for ADPT. The association between PFS and OS and cancer subtypes were assessed using Cox PH regression. Hazard ratios together with their corresponding 95% confidence intervals and Wald P values were calculated. All presented P values are two-sided. Statistical analyses were performed using R version 3.6.2 (2019 The R Foundation for Statistical Computing).

## Results

In total, 439 patients commenced eribulin for MBC at The Christie between 1st August 2011 and 31st December 2018. Patient characteristics and breast cancer history for all patients and divided by subtype are summarised in Table 1. In total, 44.0% (*n* = 193/439) of the patients had a dose reduction of eribulin, 12.5% (*n* = 55/439) were given granulocyte colony-stimulating factor (G-CSF) and 8.2% (*n* = 36/439) had both a dose reduction and G-CSF. In patients with HER2 + MBC, 7.4% (5/68) received trastuzumab concomitantly with eribulin. During the treatment period 48.3% (*n* = 212/439) patients were admitted to hospital at least one time. Reasons for hospital admissions are listed in Table 2 along with reasons for eribulin discontinuation. The median OS from the date of MBC diagnosis (MBC OS) was 41.3 months (95% CI 38.1–44.3) for all patients and differed between the biological subtypes; MBC OS_ER+/HER2-_: 46.7 months (95% CI 43.3–51.7), MBC OS_HER2+_: 48.5 months (95% CI 38.0–60.1) and MBC OS_TNBC_: 22.0 months (95% CI 18.7–25.7), *p* =  < 0.001. These results must be interpreted with caution as they are affected by survivor bias, since only those patients who survived to receive at least one dose of eribulin were included in the cohort.

**Table 1:** Patient and tumour characteristics at start of eribulin therapy, for all patients and divided by biological subtype

	All patients	ER + /HER2-	HER2 +	TNBC	*P*-value
Number of patients (%)	439 (100.0)	279 (63.6)	68 (15.5)	92 (21.0)	
DRFi, years					
Median (min–max)	53.0 (2.0–497.5)	75.9 (2.0–497.5)	37.9 (4.0–352.3)	23.0 (4.0–282.2)	< 0.001^a^
1st tertile	≤ 2.8	≤ 4.3	≤ 2.4	≤ 1.4	
2nd tertile	> 2.8–7.0	> 4.3–8.8	> 2.4–4.9	> 1.4–2.8	
3rd tertile	> 7.0	> 8.8	> 4.9	> 2.8	
De novo metastatic	75 (17.1)	47 (16.8)	14 (20.6)	13 (14.1)	
Age					
Median (range)	56 (32– 87)	56 (34–87)	56 (40–81)	54 (32–81)	0.11^a^
ECOG, n (%)					
0	137 (48.8)	88 (48.4)	21 (52.5)	28 (47.5)	0.98^b^
1	104 (37.0)	69 (37.9)	13 (32.3)	22 (37.3)	
2	37 (13.2)	23 (12.6)	6 (15.0)	8 (13.6)	
3	3 (0.1)	2 (0.1)	0 (0)	1 (0.2)	
Missing	158	97	28	33	
Metastatic sites					
Median, n	3	3	3	2	0.12^a^
Sites of metastases					
Bone	302 (68.8)	223 (79.9)	42 (61.8)	37 (40.2)	< 0.001^b^
Lung/pleura	245 (55.8)	150 (53.8)	38 (55.9)	57 (62.0)	0.39^b^
Liver	268 (61.1)	200 (71.7)	32 (47.1)	36 (39.1)	< 0.001^b^
Lymph nodes	198 (45.1)	109 (39.1)	39 (57.4)	50 (54.4)	0.003^b^
CNS	77 (17.5)	28 (13.6)	22 (32.4)	17 (18.5)	0.001^b^
Other	145 (33.0)	76 (27.2)	30 (44.1)	39 (42.4)	0.003^b^
Previous number of treatment lines for metastatic disease					
Median (range)	3 (0–11)	4 (0–11)	3 (1–11)	2 (0–5)	< 0.001^a^
Previous number of chemotherapy regimens for metastatic disease					
Median (range)	2 (0–8)	2 (0–8)	3 (1–7)	2 (0–5)	0.004^a^
≤ 2	274 (62.4)	175 (62.7)	32 (54.4)	67 (72.8)	0.004^b^
> 2	165 (37.6)	104 (37.3)	36 (52.9)	25 (27.2)	
ADPT, months					
Median (min–max)	8.0 (1.6–48.4)	9.6 (3.2–27.8)	9.6 (3.2–28.8)	6.7 (0.3–31.4)	0.031^a^
1st tertile	≤ 6.3	≤ 6.5	≤ 7.8	≤ 5.0	
2nd tertile	> 6.3–10.4	> 6.5–10.43	> 7.8–12.4	> 5.0–8.7	
3rd tertile	> 10.4	> 10.4	> 12.4	> 8.7	
NA^4^	2	2	0	1	

*ADPT* average duration of previous treatment lines, *DRFi* distant recurrence-free interval, *NA* not applicable

^a^Median Test

^b^Chi-squared Test

**Table 2:** Information on treatment and adverse events

	*n* (%)
Number of patients (%)	439 (100)
Dose reduction	
No	246 (56.0)
Yes, once	71 (16.2)
Yes, multiple	122 (27.8)
G-CSF	
No	384 (87.5)
Yes, at C1	19 (4.3)
Yes, after C1	36 (8.2)
Hospital admission^a^	
Yes	212 (48.3)
Reason(s) for hospital admission^b^	
Febrile neutropenia	54 (12.3)
Non-neutropenic infection	85 (19.4)
Eribulin-related toxicity	48 (10.9)
Other reason	106 (24.2)
Treatment-related death	
Yes	3 (0.7)
Reason for discontinuation	
Disease progression	340 (78.7)
Toxicity	24 (5.6)
Not fit for further treatment	34 (7.9)
Physician’s or patient’s choice	9 (2.1)
Other	1 (0.2)
Death	24 (5.6)
Lost to follow-up	4
NA (ongoing treatment at data cut-off)	3

^a^Number of patients admitted, not total number of admissions

^b^Patients may have had several reasons for admission

### Survival analyses

The median PFS and OS with eribulin for the whole cohort were 4.1 months (95% CI 3.7–4.4) and 8.6 months (95% CI 7.4–9.8), respectively. Outcomes were significantly inferior for those with TNBC; PFS_ER+/HER2-_: 4.6 months (95% CI 4.2–5.2); PFS_HER2+_: 3.9 months (95% CI 2.9–5.5) and PFS_TNBC_: 2.4 months (95% CI 2.1–3.0), *p* < 0.001 (Table 3) and OS_ER+/HER2-:_ 9.5 months (95% CI 8.3–11.1); OS_HER2+_: 9.2 months (95% CI 6.9–13.0) and OS_TNBC_: 5.4 months (95% CI 4.6–6.6), *p* < 0.001) (Table 4).

**Table 3:** Median progression-free survival and univariable analysis of prognostic factors for progression-free survival for all patients and divided by biological subtype

	Number of events/patients	Median PFS, months (95% CI)	Log rank *p*-value	Hazard Ratio (95% CI)	Wald *p*-value
All patients	364/439	4.1 (3.7–4.4)	< 0.001		
ER + /HER2-	229/279	4.6 (4.2–5.2)		Ref.	
HER2	57/68	3.9 (2.9–5.5)		1.03 (0.77–1.38)	0.84
TNBC	78/92	2.4 (2.1–3.0)		1.98 (1.53–2.57)	< 0.001
All patients					
DRFi, years					
1st tertile (≤ 2.8)	99/122	2.8 (2.3–4.4)	0.30	Ref.	
2nd tertile (> 2.8–7.0)	102/121	4.4 (3.7–5.0)		0.90 (0.68–1.19)	0.47
3rd tertile (> 7.0)	102/121	3.9 (3.8–5.3)		0.79 (0.60–1.05)	0.10
De novo metastatic	61/75	3.9 (3.4–4.6)		1.03 (0.74–1.42)	0.87
Linear				0.99 (0.97–1.01)	0.21
Previous chemotherapy lines, n					
≤ 2	227/274	4.3 (3.7–4.6)	0.65	Ref.	
> 2	137/165	3.9 (3.6–4.6)		1.05 (0.85–1.07)	0.66
Linear				0.94 (0.86–1.03)	0.19
ADPT, months					
1st tertile (≤ 6.3)	126/146	2.8 (2.3–3.9)	0.001	Ref.	
2nd tertile (> 6.3–10.1)	124/145	4.4 (3.8–5.0)		0.72 (0.56–0.92)	0.009
3rd tertile (> 10.1)	111/145	4.7 (4.0–5.3)		0.62 (0.42–0.69)	< 0.001
Linear				0.96 (0.94–0.98)	< 0.001
ER + /HER2-					
DRFi, years					
1st tertile (≤ 4.3)	63/78	4.4 (3.7–7.1)	0.44	Ref.	
2nd tertile (> 4.3–8.8)	65/77	5.0 (4.4–6.1)		1.06 (0.75–1.51)	0.73
3rd tertile (> 8.8)	67/77	4.6 (3.6–5.3)		1.29 (0.91–1.82)	0.16
De novo metastatic	34/47	4.4 (3.7–7.3)		1.27 (0.83–1.94)	0.27
Linear				1.01 (0.99–1.03)	0.45
Previous chemotherapy lines, n					
≤ 2	143/175	4.6 (3.7–5.3)	0.91	Ref.	
> 2	86/104	4.6 (4.3–5.3)		0.99 (0.75–1.29)	0.91
Linear				0.91 (0.80–1.02)	0.11
ADPT, months					
1st tertile (≤ 6.5)	79/93	4.1 (3.0–5.2)	0.36	Ref.	
2nd tertile (> 6.5–10.4)	80/92	5.3 (4.4–7.2)		0.80 (0.58–1.09)	0.15
3rd tertile (> 10.4)	68/92	4.6 (3.7–5.3)		0.88 (0.64–1.22)	0.45
Linear				0.98 (0.95–1.00)	0.039
HER2 +					
DRFi, years					
1st tertile (≤ 2.4)	14/18	5.0 (2.5–*)	0.62	Ref.	
2nd tertile (> 2.4–4.9)	14/17	3.7 (2.1–7.5)		1.65 (0.75–3.6)	0.21
3rd tertile (> 4.9)	14/18	3.1 (1.8–*)		1.24 (0.58–2.66)	0.58
De novo metastatic	15/15	3.9 (3.4–9.1)		1.46 (0.68–3.13)	0.33
Linear				0.97 (0.92–1.03)	0.35
Previous chemotherapy lines, n					
≤ 2	27/32	5.0 (2.3–7.5)	0.21	Ref.	
> 2	30/36	3.6 (2.5–5.5)		1.40 (0.82–2.38)	0.22
Linear				1.06 (0.87–1.28)	0.57
ADPT, months					
1st tertile (≤ 7.8)	18/23	2.9 (1.8–6.7)	0.19	Ref.	
2nd tertile (> 7.8–12.4)	20/22	2.3 (2.1–6.9)		0.71 (0.37–1.36)	0.30
3rd tertile (> 12.4)	19/23	5.0 (4.1–9.1)		0.55 (0.29–1.05)	0.071
Linear				0.95 (0.90–1.00)	0.063
TNBC					
DRFi, years					
1st tertile (≤ 1.4)	23/27	2.3 (1.8–2.6)	0.004	Ref.	
2nd tertile (> 1.4–2.8)	21/26	2.3 (1.7–4.5)		0.49 (0.26–0.92)	0.026
3rd tertile (> 2.8)	22/26	3.8 (3.0–6.0)		0.32 (0.17–0.60)	< 0.001
De novo metastatic	12/13	2.2 (1.9–*)		0.68 (0.34–1.39)	0.29
Linear				0.93 (0.85–1.01)	0.075
Previous chemotherapy lines, n					
≤ 2	57/67	2.4 (2.1–3.0)	0.48	Ref.	
> 2	21/25	2.6 (1.9–3.9)		1.20 (0.72–1.99)	0.49
Linear				0.99 (0.76–1.28)	0.92
ADPT, months					
1st tertile (≤ 5.0)	28/31	1.9 (1.5–2.7)	0.001	Ref.	
2nd tertile (> 5.0–8.7)	24/31	2.2 (1.9–4.4)		0.60 (0.35–1.05)	0.074
3rd tertile (> 8.7)	25/30	3.8 (2.6–5.0)		0.34 (0.19–0.62)	< 0.001
Linear				0.92 (0.86–0.97)	0.002

*ADPT* average duration of previous treatment lines, *DRFi* distant recurrence-free interval, *PFS* progression-free survival

**Table 4:** Median overall survival and univariable analysis of prognostic factors for overall survival for all patients and divided by biological subtype

	Number of events/patients	Median OS, months (95% CI)	Log rank *p*-value	Hazard Ratio (95% CI)	Wald *p*-value
All patients	408/439	8.6 (7.4–9.8)	< 0.001		
ER + /HER2-	255/279	9.5 (8.3–11.1)		Ref.	
HER +	64/68	9.2 (6.9–13.0)		1.08 (0.82–1.42)	0.60
TNBC	89/92	5.4 (4.6–6.6)		1.66 (1.30–2.13)	< 0.001
All patients					
DRFi, years					
1st tertile (≤ 4.3)	113/122	6.4 (5.8–9.2)	0.20	Ref.	
2nd tertile (> 4.3–8.8)	115/121	10.0 (8.5–13.5)		0.84 (0.64–1.09)	0.18
3rd tertile (> 8.8)	108/121	9.4 (7.3–12.6)		0.78 (0.59–1.01)	0.060
De novo metastatic	72/75	8.2 (6.1–10.4)		0.98 (0.73–1.32)	0.88
Linear				0.99 (0.97–1.01)	0.21
Previous chemotherapy lines, n					
≤ 2	250/274	8.8 (7.3–10.7)	0.065	Ref.	
> 2	158/165	8.2 (6.4–9.7)		1.21 (0.99–1.48)	0.065
Linear				1.02 (0.94–1.11)	0.64
ADPT, months					
1st tertile (≤ 6.5)	138/146	6.3 (5.4–7.3)	0.002	Ref.	
2nd tertile (> 6.5–10.4)	137/145	9.2 (7.7–10.9)		0.80 (0.63–1.01)	0.061
3rd tertile (> 10.4)	131/145	11.6 (9.4–14.5)		0.64 (0.51–0.82)	< 0.001
Linear				0.96 (0.94–0.98)	< 0.001
ER + /HER-					
DRFi, years					
1st tertile (≤ 4.3)	68/78	10.2 (8.3–11.6)	0.44	Ref.	
2nd tertile (> 4.3–8.8)	73/77	10.4 (7.3–15.3)		0.96 (0.69–1.34)	0.83
3rd tertile (> 8.8)	70/77	9.5 (7.1–13.4)		1.14 (0.81–1.60)	0.45
De novo metastatic	44/47	7.9 (5.9–13.9)		1.28 (0.87–1.87)	0.21
Linear				1.00 (0.98–1.02)	0.80
Previous chemotherapy lines, n					
≤ 2	156/175	10.2 (8.2–12.3)	0.14	Ref.	
> 2	99/104	8.7 (7.0–11.4)		1.21 (0.94–1.56)	0.14
Linear				1.02 (0.92–1.13)	0.70
ADPT, months					
1st tertile (≤ 6.5)	87/93	7.9 (6.8–10.6)	0.14	Ref.	
2nd tertile (> 6.5–10.4)	85/92	10.6 (8.5–13.5)		0.81 (0.60–1.10)	0.17
3rd tertile (> 10.4)	81/92	10.7 (7.5–14.8)		0.76 (0.56–1.04)	0.083
Linear				0.98 (0.95–1.00)	0.042
HER2 +					
DRFi, years					
1st tertile (≤ 2.4)	15/18	6.1 (4.4–46.5)	0.99	Ref.	
2nd tertile (> 2.4–4.9)	17/17	10.1 (6.9 –18.3)		1.05 (0.50–2.19)	0.89
3rd tertile (> 4.9)	17/18	9.3 (5.7 –18.5)		1.03 (0.49–2.14)	0.93
De novo metastatic	15/15	9.8 (6.8–15.7)		1.12 (0.53–2.36)	0.77
Linear				0.98 (0.92–1.02)	0.25
Previous chemotherapy lines, n					
≤ 2	30/32	10.1 (6.9–16.4)	0.11	Ref.	
> 2	34/36	8.5 (5.5–10.7)		1.53 (0.99–2.59)	0.11
Linear				1.09 (0.90–1.31)	0.38
ADPT, months					
1st tertile (≤ 7.8)	21/23	4.4 (3.5–10.2)	0.16	Ref.	
2nd tertile (> 7.8–12.4)	21/22	9.3 (6.9–16.9)		0.65 (0.35–1.20)	0.16
3rd tertile (> 12.4)	22/23	13.7 (8.7–16.5)		0.56 (0.31–1.04)	0.064
Linear				0.97 (0.92–1.02)	0.25
TNBC					
DRFi, years					
1st tertile (≤ 1.4)	25/27	4.4 (2.4–6.6)	0.36	Ref.	
2nd tertile (> 1.4–2.8)	26/27	5.6 (3.9–9.6)		0.83 (0.47 –1.46)	0.52
3rd tertile (> 2.8)	25/26	8.9 (4.9–15.6)		0.61 (0.34–1.07)	0.085
De novo metastatic	13/13	6.4 (3.4–*)		0.72 (0.36–1.41)	0.34
Linear				0.99 (0.92–1.06)	0.75
Previous chemotherapy lines, n					
≤ 2	64/67	6.2 (4.9–9.2)	0.72	Ref.	
> 2	25/25	4.6 (3.9–9.6)		1.09 (0.68–1.75)	0.72
Linear				1.01 (0.82–1.26)	0.89
ADPT, months					
1st tertile (≤ 5.0)	31/31	4.3 (2.5–5.4)	0.004	Ref.	
2nd tertile (> 5.0–8.7)	30/30	6.3 (3.9–9.0)		0.81 (0.48–1.36)	0.43
3rd tertile (> 8.7)	28/30	12.1 (6.5–15.9)		0.42 (0.25–0.71)	0.001
Linear				0.93 (0.88–0.97)	0.002

*ADPT* average duration of previous treatment lines, *DRFi* distant recurrence-free interval, *PFS* progression-free survival

### Prognostic factors and eribulin efficacy

Factors that were explored for eribulin efficacy are presented in Tables 3 and 4 for PFS and OS, respectively. Longer ADPT was positively associated with PFS for all patients; ADPT_1st tertile_ (≤ 6.3 months): ref, ADPT_2nd tertile_ (> 6.3–10.1 months): HR = 0.72, *p* = 0.009, ADPT_3rd tertile_ (> 10.1 months): HR = 0.62, *p* =  < 0.001 and ADPT_linear_: HR = 0.96, *p* =  < 0.001. Similar results were seen for OS; ADPT_1st tertile_ ref, ADPT_2nd tertile_: HR = 0.80, *p* = 0.061, ADPT_3rd tertile_: HR = 0.64, *p* =  < 0.001 and ADPT linear: HR = 0.96, p =  < 0.001. Longer ADPT was also positively associated with PFS and OS in the different biological subtypes, reaching statistical significance in the TNBC subgroup (Tables 3 and 4).

## Discussion

The current study represents the largest single-institution experience of the use of eribulin in MBC and includes data on outcomes for the different biological subtypes. For the whole cohort, PFS was similar but OS was somewhat shorter than in the previously published randomised trials [3, 4]. Our cohort was more heavily pretreated when compared to Study 301 [4] and 41 patients (9.3%) received ≤ 1 full cycle of eribulin and had PFS and OS of only 0.6 months (95% CI 0.5–1.2) and 1.4 months (95% CI 1.1–2.2), respectively. These patients may have been better suited for best supportive care and are unlikely to have fulfilled the eligibility criteria for clinical trials. The results of the prognostic analyses in the study were largely unchanged after exclusion of these individuals (data not shown). Other trials based on real-world data including > 100 patients report a wider spread of results for PFS (3.3–6.1 months) and OS (10.6–31.8 months) [7–14]. Differences in PFS could result from less frequent imaging or patient and tumour variability as well as differences in previous lines of MBC treatment between the cohorts. For example, Adamo et al. reported PFS of 5.5 months and OS of 31.8 months, which may be the result of 70% patients in their cohort having cancers that the authors classified as luminal A subtype [13].

As expected, the median PFS and OS in our cohort differed between the biological subtypes and were inferior in those with TNBC (Tables 3 and 4). Importantly, the outcomes for patients with HER2 + disease may not be comparable to present day worldwide expectations for two reasons. Firstly, a significant proportion of these patients did not receive HER2 dual-blockade as 1^st^-line metastatic treatment, which is known to significantly increase OS [15]. Secondly, at the time of this project the National Institute for Health and Social Care Excellence (NICE) stipulated that only two lines of anti-HER2 therapy could be used in the metastatic setting. Therefore, most patients with HER2 + cancers only had two lines of anti-HER2 therapy unless additional lines were received in the context of clinical trials or private care. The vast majority did not have anti-HER2 treatment concomitant with eribulin, which remains the treatment paradigm within the National Health Service (NHS) in England.

Based on the pooled data from EMBRACE and Study 301 reported by Cortes et al., the median OS was longer for patients having ≤ 3 previous chemotherapy lines [16]. This may be expected as OS is generally longer for patients who are early into their disease. However, we did not demonstrate significant differences in OS between patients having ≤ 2 vs. > 2 previous chemotherapy lines for MBC, nor for ≤ 3 vs. > 3 previous chemotherapy lines (data not shown for the latter). Instead, we hypothesised that it was important to not only assess the number of previous treatment lines but also to take into consideration their duration as a surrogate for efficacy. We therefore defined the variable ADPT. We found longer ADPT to be associated with better outcomes in all patients, both with regards to PFS and OS. Similar results were demonstrated for all biological subgroups, but the strongest correlation was found in TNBC. This is perhaps the case as prior lines in those with TNBC will have been chemotherapy regimens whereas in those with ER + /HER2- and HER2 + cancers, endocrine therapies and HER2-targeted agents will have contributed significantly with potentially non-overlapping mechanisms of resistance.

This study has several strengths. It is the largest single-institution real-world eribulin study, also reporting on outcomes for each biological subtype. The data were collected by review of medical records rather than register data and to our knowledge; we are the first to report on the importance of the duration of previous treatments. Although ADPT is not an established endpoint, our results indicate that the duration of prior therapy is important when predicting the benefit of chemotherapy in a palliative setting, rather than the number of previous chemotherapy lines per se. For patients living with MBC it may be very useful to know what to expect from a particular choice of therapy. For example, according to our data, a patient with TNBC and an ADPT of 3 months can only expect a PFS on average of less than 2 months and an OS of about 4 months. In contrast an ADPT in the upper tertile, of say 10 months, would predict a doubled PFS and an OS of over a year. This approach would be useful to help not only decide whether to accept the offer of further therapy but also for future planning including end of life care.

The study also has limitations. With all real-world studies, it is difficult to account for all possible confounding factors that may influence the results. The annotations in the medical records were not considered sufficiently comprehensive to present data on adverse events, except for hospitalisation. Performance status and comorbidities were relatively poorly documented in comparison to clinical trial annotations. In addition, our time frame of 7 years allowed for variability of available treatment within each biological subtype, which may have affected the outcome of PFS and OS in our cohort.

In conclusion, by introducing the variable ADPT, we show that patients who have a longer exposure to previous treatment lines have better outcomes on eribulin and this was particularly evident for patients with TNBC. This composite measure has clinical utility for patient information and decision-making at a difficult point in their metastatic journey and should be validated in additional cohorts.

## Data Availability

Data are available on request to the corresponding author.
